# Association between Relative Handgrip Strength and Chronic Lower Back Pain: A Nationwide Cross-Sectional Analysis of the Korea National Health and Nutrition Examination Survey

**DOI:** 10.3390/ijerph182010770

**Published:** 2021-10-14

**Authors:** Sungwoo Choi, Sangun Nah, Haedong Jang, Jieun Moon, Sangsoo Han

**Affiliations:** 1 Department of Emergency Medicine, Soonchunhyang University Bucheon Hospital, Bucheon 14584, Korea; csw3613@naver.com (S.C.); potter325@naver.com (S.N.); 2 Department of Orthopedic Surgery, Soonchunhyang University Bucheon Hospital, Bucheon 14584, Korea; khaki00@schmc.ac.kr; 3 Clinical Trial Center, Department of Biostatistics, Soonchunhyang University Bucheon Hospital, Bucheon 14584, Korea; moon6188@schmc.ac.kr

**Keywords:** low back pain, hand strength, sarcopenia

## Abstract

Lower back pain (LBP) is the most common musculoskeletal complaint and significantly reduces the quality of life. Muscle weakness is a major cause of LBP. Handgrip strength (HGS) is widely used to determine general muscle strength. Relative HGS (RHGS) incorporates body mass and provides a more accurate estimation of muscle strength and health status. We analyzed the data from Korea National Health and Nutrition Examination Survey to investigate the association between RHGS and chronic LBP. We identified 14,930 participants and excluded a total of 9553 participants with age < 50 years, with incomplete LBP information, and with incomplete HGS information. We analyzed the data of 5377 participants: 1086 (20.19%) with chronic LBP and 4291 (79.8%) without chronic LBP. Multivariate regression analysis with adjustment for covariates identified an association between weak RHGS and chronic LBP (odds ratio 1.31; CI 1.07–1.61; *p* = 0.01). This article demonstrated a significant association between RHGS and chronic LBP. Therefore, RHGS should be measured for patients with chronic LBP.

## 1. Introduction

Lower back pain (LBP) is the most common musculoskeletal complaint worldwide and significantly reduces the quality of life [1,2]. Risk factors for LBP include advanced age, sex, obesity, smoking, lack of exercise, trunk muscle weakness, and sitting time [1,3,4,5]. Among them, the trunk muscle is considered to be an important factor in LBP as it supports the loading applied to the body and protects the spinal structure. [5]. Many tests can be used to measure trunk muscle strength and mass, including imaging examination techniques (such as magnetic resonance imaging, computed tomography, and dual-energy X-ray absorptiometry), bioimpedance analysis, and biochemical analysis [6], which are complicated as well as expensive. Therefore, the assessment of handgrip strength (HGS) is widely used. HGS, the maximum static grip strength applied by the hand to squeeze a dynamometer, is an affordable and simple method to measure the general muscle strength [7].

HGS is used to diagnose sarcopenia and to assess general health, nutrition, disability, and hand function [8,9,10]. Additionally, HGS can be considered as an indicator of total muscle strength [11]. A previous study confirmed that low handgrip strength is associated with chronic LBP among females aged ≥ 50 years old [12]. In addition, HGS is associated with the surgical prognosis of vertebral fracture and spinal deformity [13,14]. However, HGS should be carefully interpreted, as the cut-off values vary by age, sex, ethnicity, and study population [7,15]. Therefore, relative handgrip strength (RHGS) can be used instead of HGS. This accounts for account individual differences such as body weight and mass. RHGS provides accurate information about muscle strength and health status. A recent study identified the associations of RHGS with cardiovascular and musculoskeletal diseases and disability [16,17,18,19]. In this study, therefore, we evaluated the relationship between chronic LBP using HGS that is an affordable and simple method, and RHGS that reflects individual differences.

## 2. Materials and Methods

### 2.1. Study Design

This is a nationwide cross-sectional analysis study of the Korea National Health and Nutritional Examination Survey (KNHANES). KNHANES is an annual survey program conducted by the Korea Centers for Disease Control and Prevention (KCDC) to evaluate the health and nutritional status of South Korean civilians using a nationwide, stratified, clustered, multistage, and random sampling methods. KNHANES includes different individuals each year, selected based on their age, sex, and residential area. We have conducted this study in accordance with the STROBE statement (Supplementary Table S1).

### 2.2. Data Collection

This survey randomly selects households from those who registered in the Population and Housing Census. The survey is conducted by experts and consists of a health survey, physical examination, and dietary survey [20]. In this study, the data from KNHANES 2014 (VI-2) and 2015 (VI-3) were analyzed. We analyzed data from participants of VI-2 and VI-3 with age ≥ 50 years and chronic LBP, as the KNHANES did not evaluate chronic LBP of those aged under 50 years. Additionally, HGS was measured among participants aged ≥ 10 years in VI-2 and VI-3. Participants with no hand, paralysis of hands, bandage or cast on hands or fingers, surgery on hands or wrist within 3 months, and pain or stiffness in hands or wrist within one week were excluded. We also excluded participants with missing data regarding chronic LBP or HGS.

### 2.3. Definition of Chronic Lower Back Pain

Chronic LBP was defined as an affirmative reply of participants for “whether you have experienced lower back pain for more than 30 days in the past 3 months.” 

### 2.4. Measurement of Handgrip Strength

The measurement was conducted at four Mobile Examination Centers (MEC) nationwide by four experienced and trained medical staff. MECs are equipped with standardized examination environment and equipment [21,22]. HGS was measured thrice in right and left hands using a digital handgrip dynamometer (TKK 5401, Takei Scientific Instruments Co., Ltd., Tokyo, Japan). Experienced medical staff instructed participants to grip the dynamometer as strongly as possible at 90° to the handle. Next, the measurement was repeated in a standing position with the arm in abduction. There was an interval of at least 30 s between each trial, and the highest recorded value was used for analysis [23]. RHGS was calculated by dividing the maximum HGS of the dominant hand by body mass index (BMI) [24]. Using the 50th percentile as a cut-off for males and females, the participants were divided into two groups, weak and strong HGS groups.

### 2.5. Demographic, Health-Related, and Social Variables

Data on age, sex, weight, height, BMI, sleep duration, alcohol use, smoking, education, occupation, household income, physical activity, and comorbidities were surveyed using questionnaires and interviews.

For the duration of sleep, participants were asked, “How many hours do you usually sleep a day?” Participants were divided into non-/ex-smokers and current smokers. Alcohol consumption was divided into ≤ 1 drink per month, 2 drinks per month to 3 drinks per week and ≥ 4 drinks per week. The level of education was categorized into elementary school, middle school, high school, and university education. The occupations of participants were classified into unemployed, office work/sales and services, agriculture, forestry and fishery, machine fitting, and simple labor [25]. Household income was divided into quartiles. Physical activity was defined as moderate-intensity aerobic exercise for ≥2 h and 30 min per week, or high-intensity aerobic exercise for ≥1 h and 15 min per week [26]. Comorbidities documented in this study included hypertension, diabetes mellitus, dyslipidemia, stroke, angina, myocardial infarction, arthritis, and malignancy.

### 2.6. Statistical Analysis

We compared the general characteristics of participants with and without chronic LBP. S chi-square test was used for categorical variables and Student’s *t*-test was used for comparison of continuous variables. Multiple logistic regression analysis was performed to evaluate the association between chronic LBP and HGS using three models (model 1: no adjustments; model 2: adjustments for age and sex; model 3: adjustments for age, sex, obesity, smoking, alcohol use, education, occupation, income, sleep duration, physical activity, and comorbidities). Odds ratios (ORs) were calculated with 95% confidence intervals (CIs). Statistical analyses were performed using IBM SPSS Statistics software (version 26.0; IBM Corp., Armonk, NY, USA). A *p*-value < 0.05 was considered statistically significant. To exclude bias, sampling weights were applied.

## 3. Results

**Figure 1:**
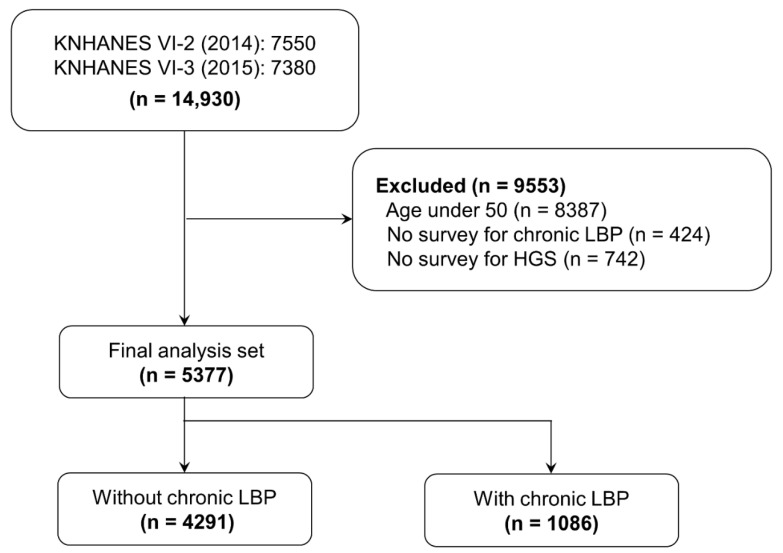
Flow of participants in 2014–2015 Korea National Health and Nutrition Examination Surveys.

There were 7550 and 7380 participants in VI-2 and VI-3, respectively. Of the total 14,930 participants, 8387 were aged < 50 years, 424 had missing LBP information, and 742 had missing HGS information. After exclusion of these participants, 5377 participants were included for the analysis in this study. Chronic LBP was present in 1086 (20.19%) and absent in 4291 (79.8%) participants (Figure 1).

### 3.1. General Characteristics of Participants with Lower Back Pain

HGS was 24.96 ± 8.4 kg and 29.68 ± 9.54 kg in those with and without chronic LBP, respectively (*p* < 0.001). The 50th percentiles for HGS in men and women were 36.7 kg and 22.5 kg, respectively. Using these values as cut-off, the HGS was strong in 427 (39.32%) and 2253 (52.51%) participants with and without chronic LBP, respectively (*p* < 0.001). The HGS was weak in 659 (60.68%) and 2038 (47.49%) participants with and without chronic LBP, respectively (*p* < 0.001). The RHGS were 1.04 ± 0.37 kg and 1.25 ± 0.41 kg in those with and without chronic LBP, respectively (*p* < 0.001). The 50th percentiles for RHGS in men and women were 1.53 kg and 0.93 kg, respectively. Using these values as cut-off, the RHGS was strong in 417 (38.4%) and 2269 (52.88%) participants with and without chronic LBP, respectively (*p* < 0.001). The HGS was weak in 669 (61.6%) and 2022 (47.12%) participants with and without chronic LBP, respectively (*p* < 0.001). 

Patients with and without chronic LBP differed significantly in terms of age, sex, height, weight, BMI, duration of sleep, smoking, alcohol use, education level, occupation, household income, physical activity, and comorbidities except malignancy (Table 1).

### 3.2. Association between Chronic Lower Back Pain and Handgrip Strength

**Figure 2:**
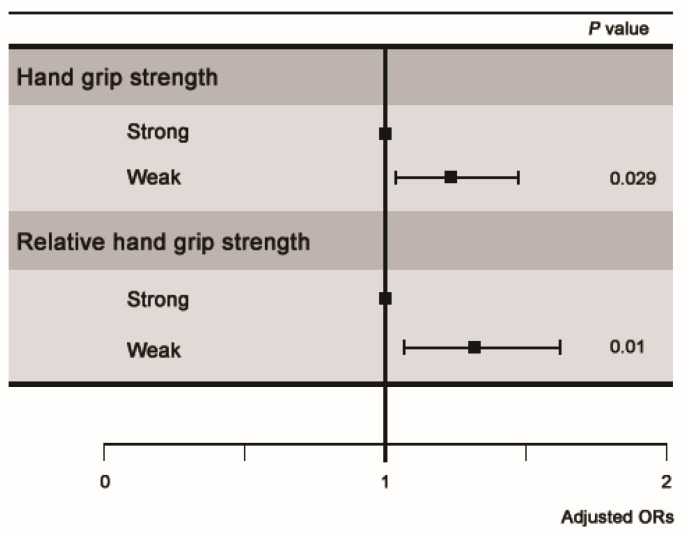
Odds for chronic lower back pain by hand grip strength and relative hand grip strength after adjusting for age, sex, obesity, smoking, alcohol use, education, occupation, income, sleep duration, physical activity, and comorbidities. OR, odds ratio.

Multivariate regression analyses were performed using three models described above. In all three models, there was a statistically significant association between chronic LBP and weak HGS (Table 2; Figure 2).

## 4. Discussion

In this study, we used the data from KNHANES to evaluate the association between chronic LBP and HGS/RHGS in the Korean population. There was a significant association between HGS and chronic LBP, even after adjusting for all confounding factors. RHGS eliminates the effects of body mass on HGS. Patients with weak RHGS had a 1.31-fold higher risk for chronic LBP compared to those with strong RHGS.

Previous studies have reported that weak trunk muscles are associated with LBP [4,5]. However, equipment for the direct measurement of trunk muscle strength is difficult to use and expensive [12]. Measuring HGS is a simple and cost-effective method to assess general muscle strength. Our study, as previous studies, confirmed a significant association between chronic LBP and HGS [12,27,28]. As HGS varies with height, weight, and BMI, it is necessary to adjust HGS for body size [7]. Therefore, RHGS (calculated by dividing HGS by BMI) is widely used. Compared to HGS, RHGS is more strongly associated with general health, sarcopenic obesity, musculoskeletal diseases, disability, and cardiovascular mortality [18,19,29]. 

Previous studies have reported an association between LBP and sarcopenia, which is an age-related loss of muscle mass and function [30,31,32]. Muscle mass is a major factor in determining muscle strength and plays a very important role in the distribution of body weight [33]. In addition, muscle weakness or loss can affect mechanoreceptors, resulting in decreased proprioceptive acuity [34]. Therefore, loss of muscle strength leads to uneven weight distribution and a decrease in proprioceptive acuity prevents correction of excessive weight on a specific body site, thereby causing musculoskeletal pain. In addition, reduced muscle mass predisposes myofibrillar proteins to breakdown by pro-inflammatory cytokines (such as tumor necrosis factor-α, interleukin-1, and interleukin-6), which increases peripheral sensitization of nociceptive afferent neurons in the muscle, as well as central sensitization to pain [35,36]. These biomechanical and neuroendocrine changes explain the results of our study. Even after adjusting for covariates in the multiple regression analyses, the odds for LBP were significantly increased with weak HGS and RHGS.

Clinicians must be aware of the decreased muscle strength in patients with LBP. They often focus solely on structural and organic problems. Although imaging and laboratory investigations are frequently performed for these patients, HGS is not usually checked. Measuring the HGS is simple, cost-effective, and quick. Sarcopenic obesity is becoming common due to recent lifestyle changes. Therefore, RHGS should be calculated to account for the body size [24]. We expect that the use of RHGS will improve the management of LBP by encouraging those with weak RHGS to engage in physical activity and exercise [37].

This study had some limitations. First, we analyzed the data from the KNHANES which was not surveyed for this study. The KNHANES was surveyed to evaluate the health and nutritional status of Korean citizen. However, this study can be considered as a representative of Korean general citizens aged ≥ 50, given that the KNHANES was conducted at the national level. Second, as it was a cross-sectional study on data from a national survey, a causal relationship between HGS/RHGS and LBP cannot be determined. However, sampling error and selection bias are expected to be minimal, as the participants were selected using clustered, multistage, and random sampling. Third, we did not evaluate the patterns and severity of LBP using quantitative scales. Therefore, we could not analyze the relationship between pain patterns or severity and HGS/RHGS. Fourth, muscle strength was determined using HGS/RHGS instead of trunk muscle strength, which is more accurate [12]. However, equipment for measuring trunk muscle strength is expensive and difficult to use. Conversely, HGS can be measured using a simple method and reflects the overall muscle strength and nutritional status. Fifth, in this study, the cut-off value of HGS/RHGS was divided into 50th percentile. The cut-off values for HGS and RHGS vary according to age, sex, country of origin, and ethnicity [38]. Additionally, there has been no standardized cut-off criteria for HGS/RHGS. Therefore, we did not use the cut-off values from previous studies. Future studies should determine the reference cut-off values for HGS and RHGS. In addition, the prevalence of LBP varies by ethnicity. We only included the Korean population, and multi-national studies are required to confirm our results in other countries. Sixth, the KNHANES only collected data on LBP from participants aged ≥ 50 years. In this study, participants only surveyed about chronic LBP were analyzed. Therefore, future studies should include patients aged < 50 years as well.

## 5. Conclusions

In this study, using data from a national health survey, there was a significant association between chronic LBP and RHGS. Participants with lower RHGS have higher odds of chronic LBP. Therefore, clinicians should evaluate muscle strength while treating patients with LBP and recognize the relationship between RHGS and chronic LBP.

## Figures and Tables

**Table 1:** General participant characteristics according to knee pain.

Variables	Without Chronic LBP (n = 4291)	With Chronic LBP (n = 1086)	*p*-Value
Age, years	63.48 ± 8.84	67.13 ± 9.14	<0.001
Sex, n (%)			<0.001
Male	2115 (49.29)	300 (27.62)	
MFemale	2176 (50.71)	786 (72.38)	
Obesity (BMI, kg/m^2^), n (%)			0.042
Underweight (<18.5)	102 (2.38)	24 (2.21)	
Normal (18.5–24.9)	2651 (61.78)	628 (57.83)	
Obese (≥25)	1538 (35.84)	434 (39.96)	
Duration of sleep, h	6.67 ± 1.43	6.38 ± 1.65	<0.001
Smoking, n (%)			0.003
Non-/Ex -smoker	3653 (85.13)	964 (88.77)	
Current smoker	638 (14.87)	122 (11.23)	
Alcohol consumption, n (%)			<0.001
None	1654 (38.55)	531 (48.9)	
≤1 drink/month	1027 (23.93)	279 (25.69)	
2 drinks/month to 3 drinks/week	1229 (28.64)	203 (18.69)	
≥4 drinks/week	381 (8.88)	73 (6.72)	
Education level, n (%)			<0.001
≤6 years	1478 (34.44)	641 (59.02)	
7–9 years	820 (19.1)	161 (14.82)	
10–12 years	1181 (27.55)	186 (17.13)	
≥13 years	812 (18.92)	98 (9.02)	
Occupation, n (%)			<0.001
Unemployed (student, housewife, etc.)	1869 (43.56)	642 (59.12)	
Office work	555 (12.93)	65 (5.98)	
Sales and services	543 (12.65)	101 (9.3)	
Agriculture, forestry, and fishery	849 (19.79)	156 (14.36)	
Machine fitting and simple labor	475 (11.07)	122 (11.23)	
Household income, n (%)			<0.001
Low	1068 (24.89)	478 (44.01)	
Low-moderate	1181 (27.53)	267 (24.59)	
Moderate-high	981 (22.86)	173 (15.93)	
High	1061 (24.73)	168 (15.47)	
Aerobic physical activity, n (%)	1046 (26.86)	175 (16.36)	<0.001
HGS, kg	29.68 ± 9.54	24.96 ± 8.4	<0.001
Strong	2253 (52.51)	427 (39.32)	<0.001
Weak **	2038 (47.49)	659 (60.68)	<0.001
RHGS, kg *	1.25 ± 0.41	1.04 ± 0.37	<0.001
Strong	2269 (52.88)	417 (38.4)	<0.001
Weak **	2022 (47.12)	669 (61.6)	<0.001
Comorbidities, n (%)			
Hypertension	1509 (35.17)	505 (46.5)	<0.001
Diabetes	541 (12.61)	192 (17.68)	<0.001
Dyslipidemia	873 (20.34)	320 (29.47)	<0.001
Stroke	136 (3.17)	72 (6.63)	<0.001
Angina	109 (2.54)	59 (5.43)	<0.001
Myocardial infarction	59 (1.37)	26 (2.39)	0.023
Arthritis	654 (16.69)	423 (38.95)	<0.001
Malignancy	123 (2.87)	34 (3.13)	0.718

Values are expressed as mean ± SD, or number (proportion). LBP: lower back pain; BMI: body mass index; HGS: handgrip strength; and RHGS: relative handgrip strength. Level of significance: *p* < 0.05. * RHGS was calculated by dividing maximum HGS from the dominant hand by BMI. ** We defined weak HGS and RHGS as < 50th percentile by sex. The 50th percentile for HGS in men and women were 36.7 kg and 22.5 kg, respectively, and for RHGS in men and women were 1.53 kg and 0.93 kg, respectively.

**Table 2:** Associations between chronic LBP and HGS and RHGS according to multiple logistic regression.

Group	Model 1			Model 2			Model 3		
	OR	95% CI	*p*-Value	OR	95% CI	*p*-Value	OR	95% CI	*p*-Value
**HGS**									
Strong	1			1			1		
Weak	1.75	1.49–2.04	<0.001	1.27	1.07–1.50	0.005	1.22	1.02–1.46	0.029
**RHGS**									
Strong	1			1			1		
Weak	1.90	1.62–2.24	<0.001	1.45	1.20–1.74	<0.001	1.31	1.07–1.61	0.01

Model 1: unadjusted odds ratio; model 2: adjusted for sex and age; and model 3: adjusted for sex, age, obesity, duration of sleep, smoking, alcohol use, education level, house income, occupation, physical activity, and medical comorbidities. HGS: handgrip strength; RHGS: relative handgrip strength; LBP: lower back pain; OR: odds ratio; and CI: confidence interval. Level of significance: *p* < 0.05.

## Data Availability

The data are available from the KCDC and Prevention database on the following webpage: https://knhanes.kdca.go.kr/knhanes/sub03/sub03_02_05.do (accessed on 5 July 2021). The data are available via this web page to anyone who meets the appropriate qualifications.

## Supplementary Materials

The following are available online at https://www.mdpi.com/article/10.3390/ijerph182010770/s1, Table S1: STROBE Statement—Checklist of items that should be included in reports of cross-sectional studies.
